# A high‐protein diet or combination exercise training to improve metabolic health in individuals with long‐standing spinal cord injury: a pilot randomized study

**DOI:** 10.14814/phy2.13813

**Published:** 2018-08-28

**Authors:** Jia Li, Keith F. L. Polston, Mualla Eraslan, C. Scott Bickel, Samuel T. Windham, Amie B. McLain, Robert A. Oster, Marcas M. Bamman, Ceren Yarar‐Fisher

**Affiliations:** ^1^ Physical Medicine and Rehabilitation University of Alabama at Birmingham Birmingham Alabama; ^2^ University of Tennessee Health Science Center College of Medicine Memphis Tennessee; ^3^ Physical Therapy and Rehabilitation Samford University Birmingham Alabama; ^4^ Department of Surgery University of Alabama at Birmingham Birmingham Alabama; ^5^ UAB Center for Exercise Medicine University of Alabama at Birmingham Birmingham Alabama; ^6^ Division of Preventive Medicine Department of Medicine University of Alabama at Birmingham Birmingham Alabama; ^7^ Department of Cell, Developmental, and Integrative Biology University of Alabama at Birmingham Birmingham Alabama; ^8^ Geriatric Research, Education, and Clinical Center Birmingham VA Medical Center Birmingham Alabama

**Keywords:** Electrical stimulation, exercise, high‐protein diet, insulin resistance, spinal cord injury

## Abstract

We compared the effects of an 8‐week iso‐caloric high‐protein (HP) diet versus a combined exercise regimen (Comb‐Ex) in individuals with long‐standing spinal cord injury (SCI). Effects on metabolic profiles, markers of inflammation, and signaling proteins associated with glucose transporter 4 (GLUT‐4) translocation in muscles were evaluated. Eleven participants with SCI completed the study (HP diet: *n* = 5; Comb‐Ex: *n* = 6; 46 ± 8 years; C5‐T12 levels; American Spinal Injury Association Impairment Scale A or B). The Comb‐Ex regimen included upper body resistance training (RT) and neuromuscular electrical stimulation‐induced‐RT for paralytic quadriceps muscles, interspersed with high‐intensity (80–90% *V*O_2_ peak) arm cranking exercises 3 days/week. The HP diet included ~30% total energy as protein (carbohydrate to protein ratio <1.5, ~30% energy from fat). Oral glucose tolerance tests and muscle biopsies of the vastus lateralis (VL) and deltoid muscles were performed before and after the trial. Fasting plasma glucose levels decreased in the Comb‐Ex (*P *<* *0.05) group compared to the HP‐diet group. A decrease in areas under the curve for insulin and TNF‐*α* concentrations was observed for all participants regardless of group assignment (time effect, *P *<* *0.05). Although both groups exhibited a quantitative increase in insulin sensitivity as measured by the Matsuda Index, the change was clinically meaningful only in the HP diet group (HP diet: pre, 4.6; post, 11.6 vs. Comb‐Ex: pre, 3.3; post, 4.6). No changes were observed in proteins associated with GLUT‐4 translocation in VL or deltoid muscles. Our results suggest that the HP‐diet and Comb‐Ex regimen may improve insulin sensitivity and decrease TNF‐*α* concentrations in individuals with SCI.

## Introduction

The escalating prevalence of metabolic disorders in individuals with long‐standing spinal cord injury (SCI) highlights the urgent need for effective interventions (Bauman and Spungen 1994, 2001). Individuals with SCI, while often relatively young, are at increased risk for developing insulin resistance and type 2 diabetes soon after the injury due to severe skeletal muscle atrophy and extreme inactivity (Bauman and Spungen 1994, 2001; Bauman et al. 1999). As a result of inactivity, body composition drastically deteriorates, including significant loss of muscle mass below the level of injury (Castro et al. 1999) and increased total fat mass (Spungen et al. 1995). In the able‐bodied (AB) population, life‐style interventions, such as diet and exercise modifications, serve as cornerstones for combatting metabolic disorders. However, only a limited number of studies have investigated the impacts of exercise (Gorgey et al. 2015a) or diet (Chen et al. 2006) on metabolic profiles among individuals with SCI, which creates a challenge for the development of optimal rehabilitation interventions.

Because individuals with SCI are not able to voluntarily activate a significant portion of total skeletal muscle, neuromuscular electrical stimulation (NMES) has been developed to facilitate exercise in this population. While aerobic or resistance exercise training with NMES has each shown some promise for improving metabolic profiles in SCI adults in a limited number of studies (Mahoney et al. 2005; Gorgey et al. 2012; Ryan et al. 2013a,b), the optimal exercise prescription is not known. Combined aerobic and resistance training (RT) have been shown to improve metabolic profiles in nondisabled individuals with type 2 diabetes when compared to aerobic training or RT alone (Sigal et al. 2007). Therefore, an exercise prescription that combines key features of resistance and aerobic exercise utilizing both upper and lower body muscles may represent an effective regimen for correcting impaired metabolic states and improving body compositions in individuals with long‐standing SCI.

Surprisingly, data on the nutritional needs of individuals with SCI who suffer from metabolic disorders are extremely limited, although one cross‐sectional analysis of individuals with long‐standing SCI showed that fat and carbohydrate (CHO) intakes are higher than recommended levels in this population (Sabour et al. 2012). Such diets are implicated in promoting poor metabolic health and adipose tissue accumulation. For example, elevated serum glucose and insulin levels after consuming a high‐CHO meal leads to increases in lipogenesis and glycogenesis (Ludwig 2003). Furthermore, increased CHO intake can influence fuel oxidation and energy expenditure, driving a shift from fat to glucose oxidation and concomitant downregulation of energy expenditure at rest (Veldhorst et al. 2010). In light of these findings, modifications to the current dietary macronutrient content may be necessary to correct poor metabolic states in individuals with SCI. Specifically, diets composed of relatively high‐protein (HP) and low‐CHO content favorably influence body composition and metabolic profiles in nondisabled individuals with or without type 2 diabetes (Layman et al. 2005; Meckling and Sherfey 2007; Wycherley et al. 2012). This positive impact is thought to be due to the satiating effect of protein that occurs despite similar or lower energy intake (Westerterp‐Plantenga et al. 1999), as well as the roles of protein in preservation of fat‐free mass (Jean et al. 2001) and insulin sensitization (Piatti et al. 1994).

To explore effective strategies for improving metabolic profiles among individuals with SCI, we aimed to compare the impacts of 8 weeks of combined exercise (aerobic + resistance) regimen (Comb‐Ex) versus HP diet. We evaluated the effects of these interventions on physiological adaptations, such as body composition, glucose homeostasis, inflammation, and lipid profiles, as well as glucose transporter 4 (GLUT4) translocation signaling pathways in paralytic and nonparalytic muscles in individuals with long‐standing SCI. We tested the overarching hypothesis that individuals with SCI who are treated with either our Comb‐Ex prescription or HP diet will both exhibit improved body compositions and metabolic profiles. However, we predicted that the improvements would be greater with Comb‐Ex as a result of the increased muscle mass and muscle metabolic capacity associated with this regimen.

## Methods

### Study participants

Individuals residing in the greater Birmingham, Alabama area were identified from a computer‐generated list of patients enrolled in the University of Alabama at Birmingham (UAB) SCI Model System. Additional recruitment was done at the UAB Spain Rehabilitation Center. This study was approved by the UAB Institutional Review Board. All participants provided written informed consent after receiving a thorough explanation of study procedures and risks and having an opportunity to ask questions.

Twenty individuals with SCI were randomly assigned to the Comb‐Ex (*n* = 10) or HP‐diet (*n* = 10) group. Eleven individuals (6 Comb‐Ex, 5 HP‐diet) completed the study. Nine participants dropped out due to lack of transportation, development of pressure ulcers, or urinary tract infections during the study. Participant characteristics are included in Table 1. Participants who completed the study had injuries ranging from the C5–T12 levels and were classified as American Spinal Injury Association Impairment Scale (AIS) class A or B. The mean age of participants who completed the study was 46.0 ± 7.8 years (mean ± standard deviation), and the mean time postinjury was 21.8 ± 6.3 years. Participants were excluded if they had any medical or health conditions that would be expected to affect interventions or testings (e.g., cardiovascular, renal, or orthopedic problems). Individuals currently on a HP diet were also excluded from participation.

**Table 1:** Baseline participant characteristics

Participant	Gender	Age	Level and AIS	Years postinjury	Body mass (kg)
High‐protein diet					
SCI 07	F	38	C5‐6/B	15	100.8
SCI 08	M	42	T2/B	25	93.4
SCI 09	M	46	T5/B	20	75.1
SCI 10	M	37	T12/A	16	90.8
SCI 11	M	44	C5‐6/B	21	148.6
Mean (SD)	N/A	41.4 ± 3.8	N/A	19.4 ± 4.0	101.7 ± 27.8
Combined‐exercise					
SCI 01	M	36	C6/B	10	90.0
SCI 02	M	58	T5‐6/A	25	100.3
SCI 03	M	55	T4/B	23	107.6
SCI 04	M	49	T6/B	31	81.7
SCI 05	M	50	T2/A	24	80.4
SCI 06	M	50	C7/B	30	65.9
Mean (SD)	N/A	49.7 ± 7.6	N/A	23.8 ± 7.5	87.7 ± 15.0

SCI, spinal cord injury; AB, able‐bodied; AIS, American Spinal Injury Association Impairment Scale.

### Interventions

#### Combined exercise

Participants performed supervised Comb‐Ex 3 days/week at the UAB Center for Exercise Medicine facility. All sessions were supervised by an experienced trainer certified by the American College of Sports Medicine or National Strength and Conditioning Association. Training consisted of a combination of exercises designed to challenge strength, power, and endurance with utilization of both upper and lower limb muscles. RT volume and intensity increased over the first four sessions for 16 upper extremity muscles. NMES knee extension training involved concentric and eccentric contractions of the knee extensor muscle group in a seated position. The full exercise prescription has been published elsewhere (Yarar‐Fisher et al. 2018).

#### HP diet

The HP diet was a weight maintenance diet that included ~30% total energy as protein (1.6 g/kg per day) with a carbohydrate to protein ratio <1.5 and fat comprising ~30% of total energy intake. All foods were provided by UAB Bionutrition Unit. The overall energy amount was estimated by multiplying the resting energy expenditure (REE, assessed via indirect calorimetry) by an activity factor. A leisure time physical activity questionnaire was used to determine the appropriate activity factor for each participant (Martin Ginis et al. 2012). To improve both dietary compliance and study retention, participants assigned to the HP‐diet group were contacted by phone 3 days/week. The full dietary prescription has been published elsewhere (Yarar‐Fisher et al. 2018).

### Outcome measurements

Before and after the 8‐week exercise training and diet program, individuals completed a battery of clinical assessments. Glucose homeostasis and insulin sensitivity were estimated, using oral glucose tolerance test (OGTT). Fasting blood draws provided samples for measurement of plasma lipid and inflammation levels. Body composition was analyzed using a dual‐energy X‐ray absorptiometry (DXA) scan, and muscle biopsies provided tissue samples for assessment of muscle metabolic signaling.

#### Oral glucose tolerance test

At baseline, participants consumed their habitual diets prior to the day of OGTT. After a 10–12 hours overnight fast, each participant consumed a 75‐g oral glucose load within 5 min. Blood samples were collected immediately before and 60, 90, and 120 min after glucose ingestion for measurement of plasma glucose and insulin concentrations. Blood was immediately centrifuged, separated to collect plasma, and frozen at −80°C until analysis. Assays were performed in the Center for Clinical and Translational Science Metabolism Core. Plasma glucose assays were performed on an automated glucose analyzer (Sirrus Analyzer; Stanbio Laboratory; Boerne, TX), and plasma insulin was measured using an immunofluorescent method on an AIA‐600 II analyzer (TOSOH Bioscience, South San Francisco, CA) per the manufacturers’ instructions. Whole body insulin sensitivity (WBIS) was calculated from OGTT data, using the Matsuda Index formula shown below:

$$\text{WBIS}=\frac{10\;000}{\sqrt{\text{fasting glucose}\times \text{fasting insulin}\times \text{glucose mean concentrations}\times \text{insulin mean concentrations}}}.$$

This index correlates strongly (*r *=* *0.73, *P *=* *0.0001) with the direct measure of insulin sensitivity derived from the euglycemic insulin clamp (Matsuda and DeFronzo 1999). Blood collected prior to glucose ingestion was used to measure levels of plasma lipids (cholesterol, triacylglycerol), lipoproteins (HDL, LDL), and plasma cytokines (IFN‐*γ*, IL‐1B, IL‐6, IL‐8, IL‐10, IL‐1, and TNF‐*α*).

#### Resting energy expenditure

REE was measured after a 12‐hour fast. Measurements were performed in a quiet and softly lit room. Room temperature was maintained between 22 and 24°C. Participants lay supine on a comfortable bed, with the head enclosed in a plexiglass canopy. After resting for 15 min, REE was measured for 30 min with a computerized, open‐circuit, indirect calorimetry system with a ventilated canopy (Delta Trac II, Sensor Medics, Yorba Linda, CA). Data collected during the last 20 min were used for analysis. Oxygen uptake and carbon dioxide production were measured continuously, and values were averaged at 1‐min intervals.

#### DXA scans

Total body fat, lean mass, and android fat mass were estimated by DXA scans (Hologic QDR‐4500W, Madison, WI). Percentage of fat mass was calculated using DXA results. Participants were scanned in light clothing while lying flat on their backs with their arms at their sides. The scans were analyzed, using ADULT software version 1.33 (Lunar Radiation, Madison, WI).

#### Muscle biopsy and immunoblotting

Muscle samples were collected from both paralytic vastus lateralis (VL) and nonparalytic mid‐deltoid resting muscles via an established percutaneous needle biopsy procedure (Bamman et al. 2004) at baseline and week 8. Mixed muscle protein lysate was prepared using established methods in our laboratory (Mayhew et al. 2011). Briefly, muscle samples (~30 mg) were homogenized after a 15‐min preincubation in 6 *μ*L/mg muscle of ice‐cold lysis buffer containing protease and phosphatase inhibitors. Lysates were then clarified by centrifugation at 15,000*g* for 40 min at 4°C. Supernatants were stored at −80°C until use. Protein concentrations were quantified by the bicinchoninic acid technique with bovine serum albumin (BSA) as a standard. Levels of total and phosphorylated proteins associated with translocation of skeletal muscle GLUT4 were assessed. Skeletal muscle mixed protein lysates (25 *μ*g) were resolved on 4–12% SDS‐PAGE gels and transferred to Polyvinylidene fluoride membranes as previously described (Thalacker‐Mercer et al. 2010; Mayhew et al. 2011). Equal loading across lanes and equal transfer were verified by staining all gels (after transfer) with Coomassie blue and staining randomly selected membranes with Ponceau S. All gels contained samples from both Comb‐Ex and HP‐diet groups loaded in series. Primary antibodies (Abs) were purchased from Cell Signaling Technologies (Danvers, MA) and used at a 1:1000 dilution in 5% goat serum (monoclonal Abs) or 2% milk + 2% BSA (polyclonal Abs). Antibodies were directed against GLUT4 transporter (#2213S), total AMPK*α* (#2532S), phospho (Thr 172)‐AMPK*α* (#2531S), total CaMKII (#3362S), phospho (Thr 286)‐CaMKII (#12716S), total Akt (#9272S), phospho (Ser 473)‐Akt (#9271S), total AS‐160 (#2447S), and phospho (Thr 642)‐AS‐160 (#4288S). HRP‐conjugated secondary Abs were diluted 1:50,000. Bound Abs were visualized by chemiluminescent detection (SuperSignal West Dura Chemiluminescent Substrate; Thermo Scientific, Rockford, IL), using a BioRad ChemiDoc imaging system. Parameters for image acquisition, using the ChemiDoc, were constant across all membranes and predefined saturation criteria was employed for the CCD camera as previously described (Bamman et al. 2004). Band densitometry was performed using BioRad Image Lab (version 5.2.1).

### Statistical analyses

Descriptive statistics were calculated for all study variables of interest, with mean ± standard deviation reported for continuous variables. Unpaired *t*‐tests were used to compare baseline group characteristics for all continuous variables. Mixed model repeated measures analysis was used to assess the effects of group (Comb‐Ex, HP‐diet), time (baseline, week 8), and group‐by‐time interaction on the outcomes of interest (parameters for glucose homeostasis, blood lipids, plasma cytokines, and expressed protein quantities in the biopsy samples). A compound symmetry covariance matrix was assumed for these analyses. Post hoc comparisons were performed, using the Tukey–Kramer multiple comparisons test. Statistical tests were two‐sided, and *P *< 0.05 was considered statistically significant. Mixed model repeated measures analysis was performed to assess the effects of extremity (arm vs. leg), time, and their interaction on expressed protein quantities within the same groups. These comparisons allowed us to investigate whether proteins in the GLUT4 translocation signaling pathway in the arm and leg respond differently over time. Pearson correlation was performed, using the SAS PROC CORR statement to assess whether the degree of body mass loss (percentage body mass loss) was associated with changes in insulin area under the curve (AUC), Matsuda index, or TNF‐α levels in either group. Statistical analyses were performed using SAS, version 9.4 (SAS Institute, Inc., Cary, NC).

## Results

### Baseline characteristics, exercise adaptations, and adherence to interventions

Participants in the HP‐diet and Comb‐Ex groups did not differ in age and body mass at baseline (Table 1). Adherence to the exercise and diet prescription was 96% (23 out of 24 exercise sessions) and 80% (four out five participants reported that they consumed all meals), respectively. Details of exercise adaptations are published elsewhere (Yarar‐Fisher et al. 2018). Briefly, participants in the Comb‐Ex group improved peak oxygen consumption (Baseline: 13.2; Week 8: 14.6 kg/mL per minute, *P *<* *0.05) and maximum voluntary upper body strength (arm curl, overhead press, chest fly, and lateral pull, *P *<* *0.05 for all). In addition, Comb‐Ex training induced significant hypertrophy of both type I and type IIa myofibers in the deltoid muscle and hypertrophy of type IIa myofibers in the VL muscle (*P *<* *0.05). Comb‐Ex training also promoted a shift in myofiber type distribution from IIx to IIa (*P *<* *0.05).

### Body composition

**Figure 1:**
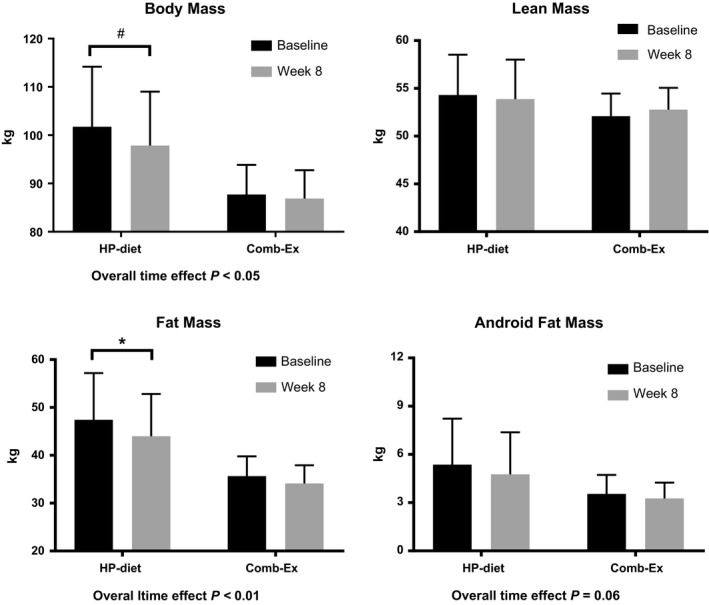
Changes in body composition in response to 8‐week interventions. Results were based on data obtained from dual‐energy X‐ray Absorptiometry (DXA) scans. *P < 0.05, ^#^ *P* = 0.10.

Changes in body composition are shown in Figure 1. Participants in both groups demonstrated a reduction in total body mass (Baseline: 95.4 ± 6.9 kg; Week 8: 93.1 ± 6.9 kg, *P *<* *0.05) and fat mass (Baseline: 41.6 ± 5.2 kg; Week 8: 39.2 ± 5.2 kg, *P *<* *0.05). Within the HP‐diet group, reduction of fat mass was significant (Baseline: 47.4 ± 7.4 kg, Week 8: 44.0 ± 7.4 kg, *P *<* *0.05). No changes were observed in total lean mass in either group. There was a trend for android fat mass reduction among participants in both groups (Baseline: 4.1 ± 0.7 kg; Week 8: 3.7 ± 0.7 kg, *P *=* *0.06).

### Glucose homeostasis

Changes in glucose homeostasis outcomes are shown in Table 2. A significant reduction in fasting glucose concentration was observed among participants in the Comb‐Ex group (*P *<* *0.05), but no change was observed in HP‐diet group. Participants had a reduction in insulin AUCs regardless of group in response to the 8‐week intervention (*P *<* *0.05). Fasting insulin concentration, Matsuda index, the homeostatic model assessment of insulin resistance (HOMA‐IR), and glucose AUC did not change in either group. Furthermore, there were no correlations between the changes in body composition (percentage of total body/total fat loss and android fat loss) and the changes in fasting glucose concentration, insulin AUC, and Matsuda index (Table 3).

**Table 2:** Intervention‐associated changes in glucose homeostasis‐related outcomes and lipid/lipoprotein and plasma cytokines profiles

Parameters	High‐protein diet			Combined‐exercise			*P* b, time	*P* c, interaction
	Baseline	Week 8	*P* a, time	Baseline	Week 8	*P* a, time		
Fasting glucose, mg/dL	95.2 ± 11.3	92.2 ± 9.6	0.79	110.3 ± 20.5	100.5 ± 13.9	0.0354	0.16	0.15
Fasting insulin, *μ*U/mL	15.7 ± 14.8	11.3 ± 14.3	0.89	16.6 ± 18.3	12.9 ± 12.4	0.91	0.36	0.93
HOMA‐IR	3.8 ± 3.4	2.4 ± 3.0	0.81	4.6 ± 5.1	3.3 ± 3.2	0.83	0.25	0.94
Matsuda index	4.6 ± 5.2	11.6 ± 9.8	0.34	3.3 ± 2.0	4.6 ± 3.8	0.98	0.15	0.32
Glucose AUC, mg/dL per minute	18,399 ± 4096	16,665 ± 3161	0.40	18,287 ± 5457	19,018 ± 4953	0.87	0.50	0.12
Insulin AUC, *μ*U/mL per minute	22,555 ± 27,937	16,083 ± 22,911	0.16	16,648 ± 17,917	12,141 ± 14,638	0.34	0.02	0.61
Cholesterol, mg/dL	168.8 ± 44.9	182.2 ± 36.7	0.82	171.8 ± 38.1	163.7 ± 27.2	0.94	0.81	0.33
Triaglycerol, mg/dL	95.6 ± 37.6	112.0 ± 39.4	0.91	120.5 ± 58.4	154.7 ± 89.8	0.47	0.16	0.61
HDL, mg/dL	46.6 ± 9.8	42.4 ± 21.3	0.73	42.0 ± 6.0	40.2 ± 7.3	0.96	0.29	0.67
LDL, mg/dL	103.1 ± 39.6	117.4 ± 45.2	0.75	105.7 ± 31.1	92.6 ± 23.3	0.75	0.96	0.19
IL‐1B (pg/mL)	0.04 ± 0.02	0.05 ± 0.02	0.92	0.04 ± 0.01	0.06 ± 0.04	0.49	0.18	0.61
IL‐6 (pg/mL)	1.2 ± 0.5	1.3 ± 0.6	0.99	1.7 ± 1.0	1.0 ± 0.32	0.20	0.23	0.13
IL‐8 (pg/mL)	8.1 ± 1.9	12.3 ± 7.3	0.25	8.9 ± 3.1	8.2 ± 1.6	0.98	0.24	0.11
IL‐10 (pg/mL)	0.27 ± 0.13	0.25 ± 0.08	0.99	0.38 ± 0.24	0.26 ± 0.22	0.31	0.17	0.34
IL‐12 (pg/mL)	0.18 ± 0.07	0.12 ± 0.0	0.49	0.18 ± 0.08	0.15 ± 0.04	0.72	0.11	0.72
IFN‐*γ* (pg/mL)	5.2 ± 2.3	3.8 ± 1.6	0.80	6.3 ± 4.3	3.9 ± 0.7	0.43	0.12	0.70
TNF‐*α* (pg/mL)	1.9 ± 0.7	1.2 ± 0.7	0.43	2.2 ± 0.4	1.4 ± 1.1	0.27	0.03	0.89

HOMA‐IR, homeostatic model assessment of insulin resistance; AUC, area under the curve.

a *P* value for within group changes.

b *P* value for main effect of time.

c *P* value for group‐by‐time interaction.

**Table 3:** Correlations between body mass/fat mass change and glucose homeostasis‐related outcomes

	Percentage body mass ∆		Percentage fat mass ∆		Android fat mass ∆	
	*r*	*P*	*r*	*P*	*r*	*P*
Fasting glucose ∆	−0.065	0.85	0.26	0.43	−0.19	0.61
Glucose AUC ∆	0.06	0.87	0.26	0.43	−0.08	0.83
Insulin AUC ∆	0.24	0.48	0.29	0.40	0.06	0.87
Matsuda index ∆	0.30	0.37	0.40	0.22	0.41	0.24

∆, change; *r*, Pearson correlation coefficient; AUC, area under the curve.

$\text{Matsuda index}=\frac{10\;000}{\sqrt{\text{fasting glucose}\times \text{fasting insulin}\times \text{glucose mean concentrations}\times \text{insulin mean concentrations}}}.$

### Lipid profile

Changes in lipid and lipoprotein levels were shown in Table 2. No changes were observed in total cholesterol, triacylglycerol, HDL, and LDL concentrations in either group.

### Inflammation profile

Changes in plasma cytokine levels are shown in Table 2. IFN‐ *γ*, IL‐1B, IL‐6, IL‐8, IL‐10, and IL‐12 levels did not change over time in either group; however, a significant reduction was observed in TNF‐*α* levels regardless of group in response to the 8‐week interventions (*P *<* *0.05).

### Skeletal muscle intracellular signaling

**Figure 2:**
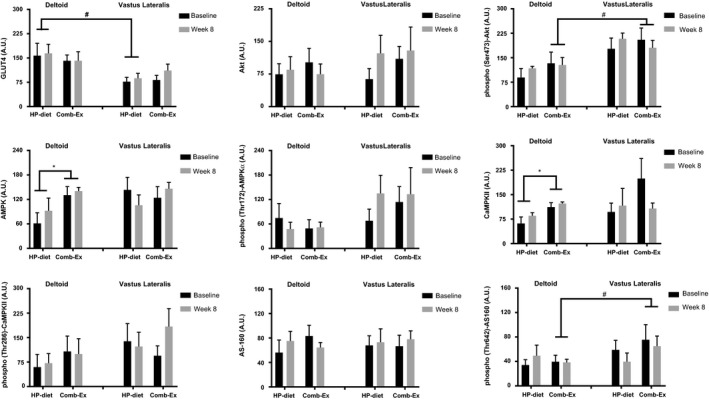
Protein levels of putative glucose transporter‐4 (GLUT‐4) signaling pathway in response to high‐protien (HP)‐diet or combined exercise (Comb‐Ex) interventions in the Deltoid and Vastus Lateralis muscle. *Significant difference between deltoid and Vastus Lateralis within the HP‐diet group. ^#^Significant difference between deltoid and Vastus Lateralis within the Comb‐Ex group.

**Figure 3:**
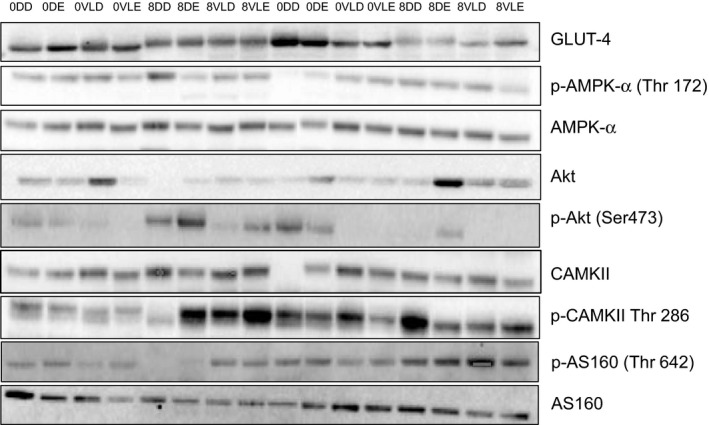
Representative immunoblots for nine studied proteins in nonparalytic deltoid and paralytic vastus lateralis (VL) in combined exercise (Comb‐Ex) versus high‐protein (HP)‐diet group. The samples were loaded in the order as shown. 0DD, week 0 deltoid‐diet; 0DE, week 0 deltoid‐exercise; 0VLD, week 0 VL‐diet; 0VLE, week 0 VL‐exercise; 8DD, week 8 deltoid‐diet; 8DE, week 8 deltoid‐exercise; 8VLD, week 8 VL‐diet; 8VLE, week 8 VL‐exercise.

There was no intervention effect on expression of putative proteins involved in GLUT4 translocation signaling pathway (Figs. 2, 3). Participants in the Comb‐Ex group had higher deltoid AMPK and CAMPKII levels compared to those in the HP‐diet group (Fig. 2, *P* < 0.05). Total levels of GLUT4 were higher in the nonparalytic deltoid muscle compared to the paralytic VL muscle in the HP‐diet group (*P *<* *0.05). In addition, there was a tendency toward higher GLUT4 levels in the nonparalytic deltoid compared to the paralytic VL in the Comb‐Ex group. Akt and AS‐160 phosphorylation was higher in the paralytic VL compared to nonparalytic deltoid muscle in the Comb‐Ex group (*P *<* *0.05).

## Discussion

Exercise regimens for individuals with SCI have been commonly studied to assess their impacts on body composition and metabolic health. However, to our knowledge, this pilot study is the first to investigate the effects of a HP diet on cardio‐metabolic health outcomes in this population. Our results suggest that consuming a HP diet or participating in a Comb‐Ex regimen may improve insulin sensitivity and decrease circulating levels of pro‐inflammatory cytokine TNF‐*α* in individuals with long‐standing SCI. No apparent resting‐state skeletal muscle signaling adaptations were observed in response to HP diet or Comb‐Ex. interventions.

Our data did not support a differential effect of HP diet or Comb‐Ex regimen on glucose homeostasis at fasting or during OGTT. However, the average fasting glucose concentrations were significantly reduced from a level that was indicative of prediabetes to nearly normal in the Comb‐Ex group. Though this finding is in agreement with those from a previous study showing reduction of glucose concentrations with RT (Mahoney et al. 2005), the interpretation warrants caution. Despite randomization, there were more participants in the Comb‐Ex group who had type 2 diabetes mellitus (T2DM) or prediabetes compared to the HP‐diet group (*n* = 3 vs. *n* = 0) (American Diabetes, Association, 2017). Individuals with or without type 2 diabetes may respond to lifestyle interventions differently; specifically, those with T2DM tend to have greater reductions in fasting glucose than those without T2DM (Samaha et al. 2003).

We also found that although glucose AUCs did not change, insulin AUCs were reduced among all participants as a result of the interventions, suggesting an improvement of insulin sensitivity in response to the HP‐diet and Comb‐Ex regimen. Furthermore, despite nonsignificant changes in Matsuda index, the magnitude of the improvement was clinically meaningful among participants in the HP‐diet group, where the Matsuda index increased from 4.6 (borderline insulin resistance) to 11.6 (insulin sensitive). Among different AB populations, Matsuda index values between four and five have been proposed as cut‐off levels for insulin resistance (with lower values being more insulin resistant) (Radikova et al. 2006; Martinez‐Hervas et al. 2011; Gutch et al. 2015). The beneficial effects of HP diets on glucose homeostasis are well‐documented by previous studies among AB individuals with type 2 diabetes, or at risk for type 2 diabetes (Gannon et al. 2003; Gannon and Nuttall 2004). In our study, one of the underlying mechanisms through which the HP diet led to the apparent improvement of insulin sensitivity may be the weight loss. Despite being structured using a weight maintenance design, participants in the HP diet group lost an average of 3.9 kg (~3.8%) compared to only 1.1 kg (~1.3%) body mass in the Comb‐Ex group. Given that moderate weight loss alone is associated with improvement of fasting glucose/insulin and HbA1c concentrations (Kopp et al. 2003), it is inconclusive whether the improvement was a result of consuming a HP diet, weight loss, or a combined effect.

Chronic inflammation, such as elevated TNF‐*α* concentrations, is implicated in the development of peripheral insulin resistance (Moller 2000; Borst 2004). Due to extreme physical in activity, individuals with SCI often develop excessive ectopic adiposity (Gorgey et al. 2015b). Ectopic adipose tissues actively produce proinflammatory cytokines including TNF‐α, which are key contributors for developing systemic inflammation (Snel et al. 2012). Existing literature shows that exercise training, either an acute bout or chronic, can reduce TNF‐*α* concentrations (Rosety‐Rodriguez et al. 2014) via inhibiting TNF‐*α* synthesis (acutely) or inducing favorable changes in body composition (chronically) (Pedersen 2017). In our study, given that participants in both groups lost body mass and android fat mass (*P *=* *0.06), the decrease in TNF‐*α* concentrations may be simply due to the improved body composition.

Limited research has shown equivocal results regarding changes in lipid and lipoprotein levels with NMES exercise interventions for SCI populations. Griffin et al. (2009) showed no change in triglyceride and LDL levels and a decrease in HDL levels in response to functional electrical stimulation cycling. In contrast, NMES‐RT combined with dietary consultation (where participants were encouraged to consume a standard healthy diet containing 45% CHO, 30% fat, and 25% protein) reduced triglyceride and cholesterol/HDL ratio (Gorgey et al. 2012). In fact, the equivocal impact of exercise on lipid‐lipoprotein levels is not unique to individuals with SCI. A meta‐analysis found only small magnitudes of improvement in cholesterol, LDL, and triglyceride with aerobic exercise and inconclusive evidence for RT on lipid and lipoprotein levels among AB populations (Tambalis et al. 2009). On the other hand, diets higher in protein and lower in carbohydrate content are often found to provide benefits on HDL (Santesso et al. 2012) and triglyceride status (Santesso et al. 2012; Wycherley et al. 2012). Dietary carbohydrate provides precursors for the production of triglyceride; as a result, lower carbohydrate intake leads to reduced liver triglyceride synthesis (Kersten 2001). However, in our study, we did not observe any change in the lipid and lipoprotein profile when participants consumed the HP diet. Interestingly, the aforementioned study comparing NMES‐RT + dietary consultation versus dietary consultation alone showed significant improvements in the insulin profile and lipid metabolism (triglyceride, cholesterol/HDL ratio) only when NMES‐RT and the healthy diet regimen were combined (Gorgey et al. 2012). These results suggest that effects of exercise and diet on lipid and lipoprotein profile may be additive. In fact, among AB individuals, evidence supports such additive benefits of a HP diet and an exercise regimen on body composition and metabolic health (Layman et al. 2005).

Poor glucose tolerance in individuals with long‐standing SCI may be attributed, at least partially, to low muscle mass and low levels of muscle GLUT4, as the amount of GLUT4 is a primary factor in determining the maximal rate of glucose transport into skeletal muscle (Dohm 2002). In our previous work (Yarar‐Fisher et al. 2013), we have shown that GLUT4 protein content was lower in the paralytic VL muscle of participants with SCI compared to the nonparalytic VL muscle of AB individuals. But the basal phosphorylation states of signaling proteins (e.g., AMPK*α*2, CaMKII, and Akt) that stimulate muscle glucose uptake were generally elevated in SCI individuals compared to AB. Our current results support our earlier findings showing markedly lower baseline GLUT4 levels in paralytic VL versus nonparalytic deltoid muscle, which suggests a potential nonhomogeneous distribution of glucose‐handling capacity in paralytic versus nonparalytic muscles of individuals with SCI.

In AB individuals, GLUT4 levels have been shown to be higher in slow (Type I) compared to fast (Type IIx) muscle fibers (Daugaard et al. 2000; Gaster et al. 2001). In the present study, myofiber type distribution was assessed by myosin heavy chain isoform immunohistochemistry, and results were reported elsewhere (Yarar‐Fisher et al. 2018). Participants in the HP‐diet and Comb‐Ex groups combined had 16% Type I fibers, 40% Type IIa fibers, and 43% Type IIx fibers in the paralytic VL muscle versus 54% Type I fibers, 35% Type IIa fibers, and 10% Type IIx fibers in the nonparalytic deltoid muscle at baseline. Lower GLUT4 in paralytic muscles may be due to the greater proportion of fast muscle fibers in paralytic VL versus nonparalytic deltoid muscles, which may result in impaired glucose‐handling capacity under exercise or insulin‐stimulated conditions in paralytic muscles. These results suggest that early prevention of these deleterious fiber‐type adaptations may potentially be more effective than attempting to reverse changes years after SCI.

Akt and AS‐160 have been identified as two of the key signaling proteins that regulate glucose uptake via GLUT4 translocation upon insulin stimulation (Karlsson et al. 2005) or muscle contraction (Cartee and Wojtaszewski 2007). The presence of higher basal phosphorylation of Akt and AS‐160 in the absence of contraction‐ or diet‐mediated induction of signaling in paralytic VL muscle in the current study suggests an attempted compensatory response to low muscle GLUT4 protein levels. To our surprise, no significant change was noted in the levels of total or phosphorylated glucose uptake signaling proteins in VL or deltoid in response to Comb‐Ex in this study. The regulation of these signaling processes is of course influenced by mode, intensity, and volume of exercise/contraction. Aerobic‐NMES combined with NMES‐RT, which is perhaps the most extreme metabolic stressor for chronically deconditioned paralytic muscle, may be needed to increase the expression of glucose uptake signaling proteins. In addition, given that nonparalytic deltoid muscle is accustomed to daily contractions of varying intensities via every day activities (e.g., bed transfers) in individuals with SCI, a higher intensity or duration of muscle activity may be needed to improve muscle glucose uptake signaling in nonparalytic muscles.

Our pilot study has several limitations. First, the study was limited to five and six participants in the HP‐diet and Comb‐Ex group, respectively. As a result, there may not be sufficient power to detect significant changes for some of the parameters. Second, even though weight maintenance was intended, participants in the HP‐diet group lost a moderate amount of body mass. Therefore, it is unclear whether the observed improvements were a result of the HP diet or body mass loss. The decrease in body mass may be a result of water loss (due to the low content of sodium and carbohydrate in their diet) (Rabast et al. 1981) or incomplete consumption of prescribed diet (due to protein's satiating effect), resulting in negative energy balance (Soenen and Westerterp‐Plantenga 2008). Future studies are warranted to assess the direct effects of HP intake on metabolic health in individuals with SCI. Third, information regarding participants’ habitual physical activity and dietary intake was not collected. Whether our interventions elicited substantial changes in participants’ diet or physical activity behaviors cannot be confirmed. Lastly, our study did not have a no‐intervention control group; therefore, it is unknown how the reported outcomes would change over time without a diet or exercise intervention.

In conclusion, our findings suggest that a HP diet or Comb‐Ex regimen may improve insulin sensitivity and decrease pro‐inflammatory TNF‐*α* levels in individuals with SCI. It paves the way for defining the most effective nutritional or exercise interventions for individuals with SCI. For future studies, it is of interest to investigate whether the combination of HP diet and exercise would offer greater improvements than either intervention alone.

## Conflict of Interest

None declared.
